# A large French family with *TGFBR2* pathogenic variant: illustration of variability

**DOI:** 10.1186/s13023-025-04124-1

**Published:** 2025-11-27

**Authors:** Ludivine Eliahou, Olivier Milleron, Skerdi Haviari, Florence Arnoult, Nadine Hanna, Pauline Arnaud, Fadima Toure, Sabrine Jadoui, Arienne Mirmiran, Catherine Boileau, Guillaume Jondeau

**Affiliations:** 1 Centre National de Référence pour le syndrome de Marfan et apparentés, maladies aortiques rares, VASCERN HCP, Service de Cardiologie, Hôpital Bichat, 46 rue Henri Huchard, Paris, 75018 France; 2 Université Sorbonne Paris Nord, IAME, INSERM, Paris, 75018 France; 3 Département Epidémiologie Biostatistiques et Recherche Clinique, AP-HP, Hôpital Bichat, Paris, 75018 France; 4 Université Paris Cité, Paris, France; 5 Département de génétique AP-HP, Hopital Bichat, Paris, France; 6 INSERM U1148, LVTS, Hopital Bichat, Paris, France

**Keywords:** *TGFBR2*, Genotype-phenotype correlation, Modifier genes

## Abstract

**Aims:**

To report aortic events in a large family carrying a variant in the *TGFBR2* gene.

**Methods:**

Since 1990 up to 2024, we have conducted a longitudinal clinical study of a large single family comprising 63 members across four generations who carry the same *TGFBR2* pathogenic variant. We assessed the incidence of aortic events and prophylactic surgery, as well as life expectancy.

**Results:**

Over a follow-up, 21 patients died (33% of the population), of whom ten were related to a dissection (48%). Eight patients underwent prophylactic aortic root surgery (13%). Over the generations, there is an increase in life expectancy and a decrease in the likelihood of aortic dissection (p < 0.001), but no significant change in the combined endpoint of surgery, aortic dissection or death (p = 0.168). The type of prophylactic surgery has also evolved over the years from mechanical Bentall surgery to valve-sparing surgery. The variability in the age at onset of aortic events reflects the broad phenotypic spectrum of aortic disease associated with this variant.

**Conclusion:**

Across four generations, improved diagnosis, prophylactic surgery, and surgical techniques were associated with reduced dissection rates and increased survival, despite marked intra-familial variability in aortic disease severity among carriers of the same *TGFBR2* pathogenic variant.”

## Introduction

Aneurysms of the ascending aorta are usually asymptomatic but potentially fatal due to the risk of aortic dissection. Some are of genetic origin (autosomal dominant transmission), and family screening may improve the prognosis of these patients. Pathogenic/likely pathogenic (P/LP) variants can affect genes coding for different pathways: extracellular matrix, smooth muscle cell contractile proteins and TGF-β pathway [including variants in the TGF-β receptor 2 gene, *TGFBR2*]) [1] ORPHA:60030. The initial recognition of the role of the TGF-β pathway, and in particular *TGFBR2* was made possible by linkage analysis in a large French family for which follow-up data are reported here[2].

In patients with P/LP variants in the *TGFBR2* gene, great variability is observed both in the severity of the aortic and in extra-aortic vascular features, and the presence and extent of extra-aortic involvement. Some patients with these variants have aggressive aneurysms with early dissection, which are part of the Loeys Dietz Syndrome. Others have non-syndromic late-onset aneurysms, and still others are asymptomatic carriers. This variability is illustrated by a mean age at death at 26 years in the syndromic population described by Loeys et al. [3], and a survival rate close to that of classic Marfan syndrome linked to the *FBN1* gene in a less syndromic population reported in an international study [4, 5]. This observation has led to much discussion about the significance of Loeys Dietz syndrome[6].

This variability may be related to phenotype-genotype correlation, meaning that a particular variant may be associated with a more (or less) severe phenotype [7, 8]. However, the variability is also observed within a given family, i.e., in patients carrying the same *TGFBR2* P/LP variant, which is even more striking. However, the limited number of patients with a specific variant makes it difficult to evaluate this variability. Here, we take advantage of this large family, to describe the clinical phenotype of the different affected members.

## Materials and methods

The first members of this family have been recognised initially as having “Marfan syndrome” in 1990 and have been followed up since then. The responsible P/LP variant in this family is causing the synonymous amino acid substitution, Q508Q (1524 G→A) located in *TGFBR2* gene [2] This variant was classified as pathogenic (class five) according to ACMG classification.

All subjects originated from the French National Reference Centre for Marfan Syndrome and related disorders and gave their written informed consent for participation in this clinical and genetic study in agreement with the requirements of French regulations. The personal protection committee of sud-Méditerrannée I approved this study under the committee’s reference number 21.04393.000082. All the clinical information available from the medical records was collected and supplemented by interviews with the treating physicians, as the various family members were followed in different places in France and other countries.

A family member was considered affected if they carried the familial P/LP variant, regardless of their clinical features. They were also considered affected if they had experienced an aortic event such as prophylactic aortic root surgery or aortic dissection, either type A or B. Additionally, obligate carriers were classified as affected, even if their genetic status was unknown. Sudden death was considered secondary to a type A dissection if the age was less than 50 years unless proven otherwise. Aortic events included aortic dissection and surgery. Parents at risk are family members with a first-degree relative affected.

Many patients were seen at least once at the reference centre for Marfan syndrome and related disorders, which was initially based at the Ambroise Paré Hospital in Boulogne-Billancourt and moved to the Bichat Hospital in Paris in 2007. There, the patient’s evaluation included echocardiography with measurement of aortic diameters at various levels, including the sinuses of Valsalva, an ECG, an ophthalmological evaluation, and a skeletal and dermatological evaluation.

For statistical analysis, only the first event (aortic dissection or prophylactic aortic surgery) has been considered. Fine-Gray models were used to account for competing risks. Stacked Aalen-Johansen estimators are shown on graphs. The calculations were performed using the survival package in R.

## Results

### Population (Fig. 1)

**Fig. 1:**
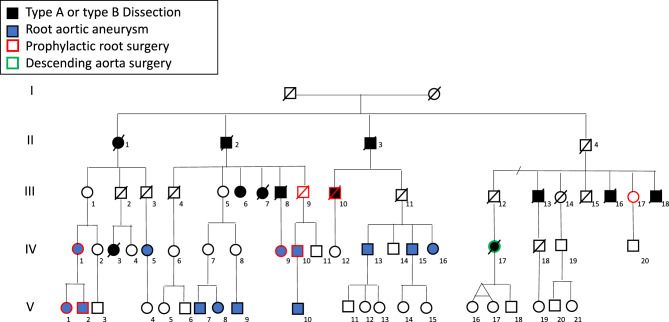
Pedigree of the family, including only affected members. Sixty-three patients across four generations have been identified; patient III:6: she had a bentall surgery after aortic dissection. Patient III:7: she had ruptured abdominal aneurysm followed by endoprosthesis surgery. She finally died of endocarditis. Patient III:17: she underwent surgery for dilatation of the ascending aorta, but the type of surgery remains unknown. Patient IV:17: she first underwent a type B dissection followed by surgery of the descending aorta and, a few years later, a type a dissection that led to her death

Sixty-three members (32 males and 31 females) were recognised as affected over four generations out of 151 at risk family members (42%). Four out of five (80%) at risk family members from generation II, 18 out of 32 (56%) from generation III, 20 out of 64 (31%) patients at risk from generation IV and 21 out of 50 (42%) patients at risk from generation V.

### Aortic events

**Fig. 2:**
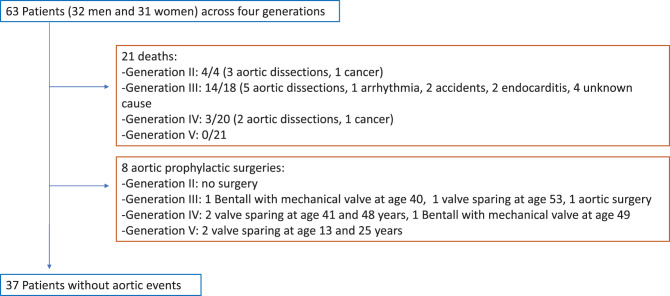
Aortic event risk flow chart

A flow chart summarising the aortic events is shown in Fig. 2. One affected woman died at the age of 40 years postpartum. This was not considered an aortic dissection and therefore not included in the flowchart because the cause and manner of death were unclear and were reported to be related to obstetric problems.

**Fig. 3:**
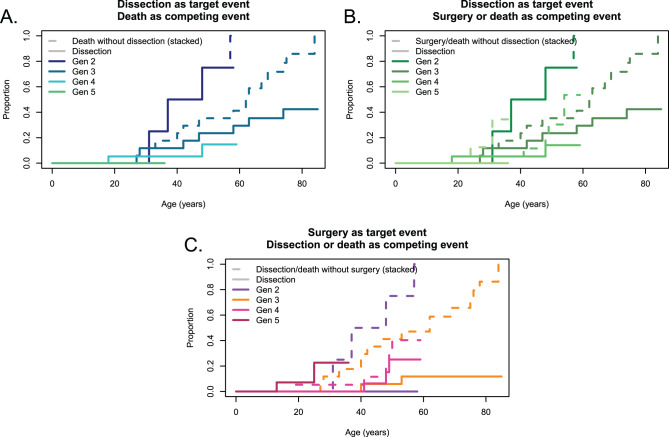
Stacked cumulative incidence curves. Life expectancy has increased over the generations (panel a, *p* < 0.001), likelihood of aortic dissection has decreased (panel a) even when accounting for surgery (panel B, *p* < 0.001), which is performed earlier (panel C, *p* < 0.001). Each color corresponds to a generation. Lines are stacked Aalen-Johansen estimators, with competitors (dotted) stacked above events of interest (solid) for each generation. Overall p-values were obtained by a Wald test on a fine & gray model with separate categories for each generation
- Eight preventive surgeries of the aortic root at a mean age of 36.6 years, range 18-53 years, three in generation III, three in generation IV and two in generation V.

Aortic events affected 20 patients and included:

- Twelve aortic dissections (Fig. 3A, 3B), at a mean age of 43 years, range 18-84 years. Seven type A, one type B, four unknowns; Three in generation II (75% of the generation II); seven in generation III (39% of the generation III) et two in generation IV (10% of the generation IV). Interestingly, aortic root dissections were observed only in untreated and undiagnosed patients whereas the type B dissection was observed also during follow-up.

Of note, aortic risk was similar in men (nine aortic events/32) and women (10 aortic events/31) in this family.

### Deaths

**Fig. 4:**
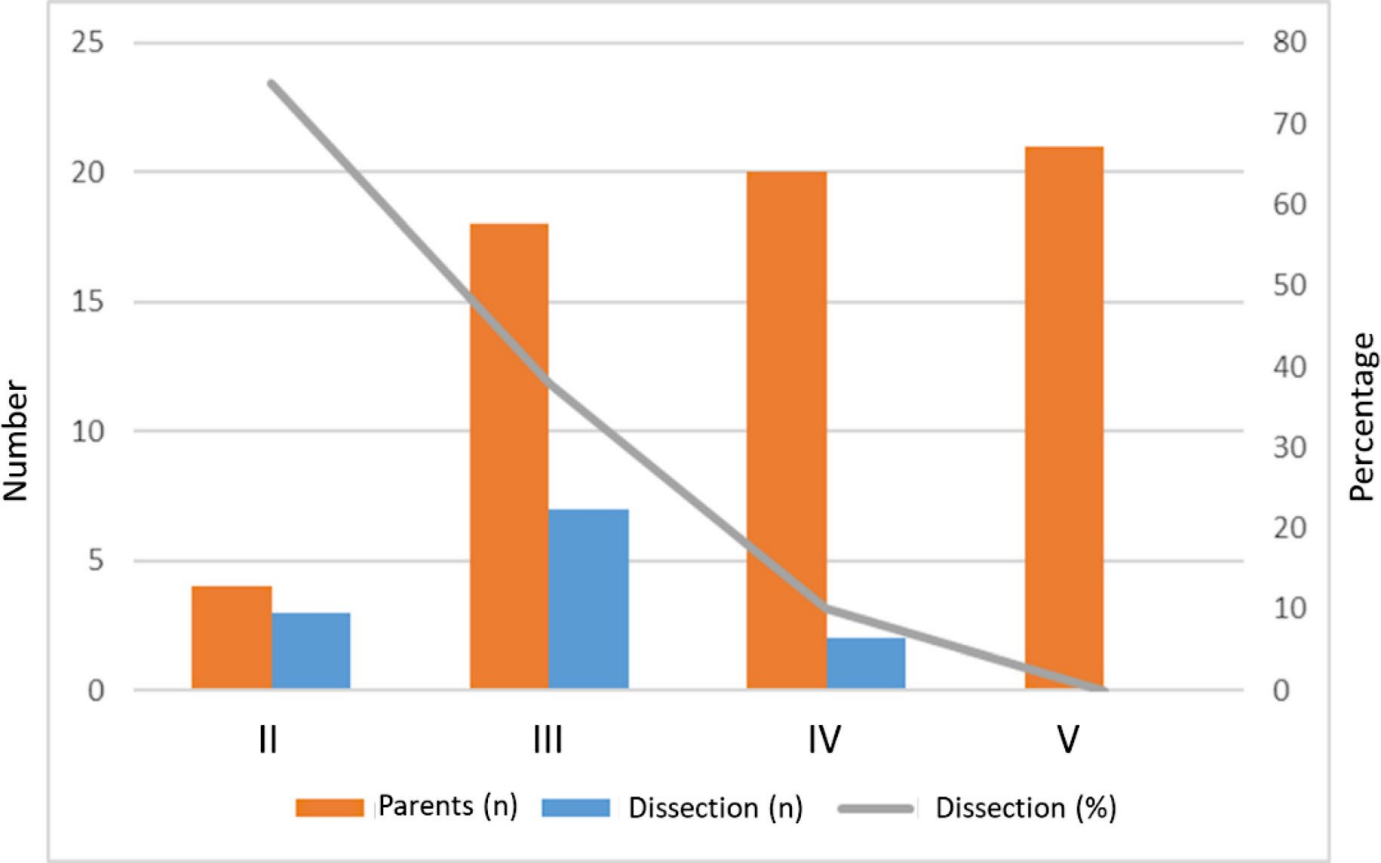
Evolution of aortic dissections over generations

Survival curves are shown in Fig. 3. Over the generations, life expectancy increased (Fig. 3A) while the risk of aortic dissection decreased (Figs. 3A and 4) even when adjusting for competing surgeries (Fig. 3B), which were performed earlier (Fig. 3C). As a result, the combined endpoint including prophylactic aortic surgery, aortic dissection or death does not vary significantly, consistent with the fact that prophylactic aortic surgery prevents aortic dissection and death but does not alter the course of aortic disease, which remains stable across generations.

Since this manuscript was written, another type B dissection has occurred in an 81-year-old woman. The aortic root diameter was normal (35 mm) at the time of the event. She was not on beta-blocker treatment due to poor tolerability. She died of aortic rupture at the age of 82, having refused surgery.

### Aortic diameters

Aortic diameters and Z-scores were available for 35 and 22 patients, respectively. The largest aortic diameter was observed at the level of the aortic root in all patients. The z-scores derived from the Campens nomogram [9] were similar in all age groups of unoperated affected subjects (Table 1). A bicuspid aortic valve was found in 3/38 (7%) of the patients.

**Table 1:** Measurement of aortic diameter in different age groups and z-scores derived from the Campens nomogram

	N	Zscore	Aortic diameter (mm)
0–20 years	6	2.24	31.5 ±7
20–30 years	3	3.2	39.5 ± 2.36
30–40 years	5	2.7	37.8 ±5.3
40–50 years	7	2.7	41.1 ± 6.7
> 50 years	1	4.3	41 ± 8.2

### Therapy

Of the 35 patients who were followed up, 25 were taking beta-blockers, two were taking bradycardia-inducing calcium channel blockers due to poor tolerance of beta-blockers, and eight were not taking any treatment. No ARB was used in this family.

### Variability in severity

The youngest patient with aortic dissection was a 17-year-old girl and the oldest patient without aortic involvement was an 80-year-old woman. The total population can be artificially divided into two groups:

- Patients with severe vasculopathy, defined by a history of dissection or prophylactic surgery before the age of 40 years. It includes seven patients: five presented with type A aortic dissection at a mean age of 28 years, and two underwent prophylactic aortic root surgery (at 30 and 25 years for aortic dilatation of 50 and 53 mm); three were men and four were women. Of note, aortic root dissection was only observed in untreated and undiagnosed patients.
- Patients with mild vasculopathy, defined by no aortic event and no significant aortic dilatation (Zscore < 3) after age equal to or greater than 40 years. It includes seven patients, two men and five women, with a mean age of 50 years. Two patients belonged to generation III and five patients to generation IV. Three were treated with a BB, one with a calcium channel blocker, two patients were untreated, and the treatment of the last patient was unknown.

No extra-aortic vascular clinical events were observed in this family (including a normal brain CT scan in ten patients).

### Extra-vascular features

Skeletal features were assessed in 38 patients. Thirteen (34%) had no skeletal features. According to Ghent 1, 14 patients (37%) had mild involvement, seven (18%) had moderate involvement, and four (10%) had severe involvement. Arachnodactyly was the most common feature, present in 15 patients (39%) and pectus in 12 (32%). The other features were less common: Hyperlaxity in nine, flat feet in eight, arched palate in seven, scoliosis greater than 20° in five, dolichostenomelia in two. Protusio acetabuli was observed in three. None presented pneumothorax. Two mitral valve prolapses were found in our population. No bifida uvulae or hypertelorism were reported.

## Discussion

This large family was instrumental in demonstrating the genetic heterogeneity of what was then known as “Marfan syndrome” [10], and subsequently in recognising the role of *TGFBR2* variants in hereditary thoracic aortic disease[2].

The family is interesting for many reasons: It illustrates the variability of the phenotype associated with *TGFBR2* pathogenic variants. This family was originally described and treated as having Marfan syndrome [11], while other *TGFBR2* variants have been associated with specific phenotypic features. This has led to the description of Loeys-Dietz syndrome [12], which may be associated with severe infantile vasculopathy. Other patients present with a Marfan-like syndrome, exhibiting skeletal features similar to those observed in classic Marfan syndrome (formerly known as MFS type 2). Other patients present with isolated aortic disease, which is often discovered following aortic dissection. Furthermore, some variant carriers exhibit no symptoms or clinical features. This variability may be the result of genotype/phenotype correlations, with some variants being more severe than others, i.e., some families carrying a more severe arterial phenotype than others; some evidence is beginning to emerge that this is indeed the case [8, 13]. Our family also suggests that all the constitutive features of Loeys-Dietz syndrome may be missing in large families with a P/LP *TGFBR2* variant.

Our family also illustrates the fact that this variability can be observed within families: a similar variant can be associated with either a severe vascular phenotype or no vascular phenotype. The constitution of two groups, one comprising seven patients with unclear dilatation despite being aged 40 or over, and the other comprising seven patients with either aortic dissection or having undergone surgery before the age of 40 illustrates this variability. Another example of this variability is the occurrence of aortic dissection at the age of 17 in a woman, compared to the absence of aortic dissection in an 80-year-old woman. The disease-causing variation has a consequence on splicing, leading to premature truncating codon. The transcript analyses may identify minor abnormal transcripts in this family. One can hypothesize that the different transcripts are expressed differently between individuals, and that this may explain a part of the phenotypic variability in this family. Alternatively, this variability may be the effect of risk alleles in modifier genes. In addition to potential genetic modifiers, environmental factors such as smoking, uncontrolled hypertension and intense physical activity could contribute to the phenotypic variability observed in this family.

Unlike what is commonly observed in the literature [5], the male-to-female sex ratio for aortic events was 1 in our family, whereas such events are typically more frequent in males. The reason for this observation is unclear and may be related to the relatively small size of our population.

The present family illustrates the progress made in patient care. The number of aortic dissections decreased from generation II to generation IV, while prophylactic aortic surgery became more common. The aortic event changed from aortic dissection to prophylactic aortic root surgery (see Fig. 3). This is consistent with our previous reports on patients with Marfan syndrome, and indicates that residual dissected aorta, a major risk factor for future events, is now being avoided in these patients, unless they develop type B dissection. Additionally, the type of surgery has evolved from mechanical Bentall surgery to aortoplasty [14, 15]. This change has many advantages, as highlighted by recent comparisons of the two techniques [16, 17]. Consequently, an increase in life expectancy can be expected over time, as was observed in the present family. The present family also illustrates the importance of screening as no dissection occurred in patients with a diagnosis made, and the various dissections reported occurred before the availability of echocardiography, or in family members who were unaware of the diagnosis made in their distant relatives [18].

Our study has several limitations: Firstly, it is a retrospective study covering a long period, during which diagnostic practices evolved. Secondly, not all family members were systematically screened for extra-aortic vascular features or connective tissue signs, which could result in the underestimation of associated findings. Lastly, despite being a very large family, the number of participants remains limited.

## Conclusion

This large family has previously enabled the demonstration of genetic heterogeneity in heritable thoracic aortic diseases. It now demonstrates the clinical variability within a single *TGBFR2* variant and that Loeys-Dietz features may be less evident in certain families. Finally, advances in care are evident across generations in this family, leading to increased survival.

## Data Availability

Data are available from the authors upon reasonable request.
